# Cost-effectiveness of universal screening for chronic hepatitis B virus infection in China: an economic evaluation

**DOI:** 10.1016/S2214-109X(21)00517-9

**Published:** 2022-01-18

**Authors:** Shu Su, William CW Wong, Zhuoru Zou, Dan Dan Cheng, Jason J Ong, Polin Chan, Fanpu Ji, Man-Fung Yuen, Guihua Zhuang, Wai-Kay Seto, Lei Zhang

**Affiliations:** a China–Australia Joint Research Centre for Infectious Diseases, School of Public Health, Xi’an Jiaotong University Health Science Centre, Xi’an, China; b Department of Family Medicine and Primary Care, Li Ka Shing Faculty of Medicine, The University of Hong Kong, Hong Kong; c Department of Medicine and State Key Laboratory of Liver Research, The University of Hong Kong, Hong Kong; d Department of Family Medicine and Primary Care, The University of Hong Kong–Shenzhen Hospital, Shenzhen, China; e Department of Medicine, The University of Hong Kong–Shenzhen Hospital, Shenzhen, China; f Central Clinical School, Faculty of Medicine, Monash University, Melbourne, VIC, Australia; g WHO West Pacific Regional Office, Manila, Philippines; h Department of Infectious Diseases, Second Affiliated Hospital of Xi’an Jiaotong University, Xi’an, China; i Artificial Intelligence and Modelling in Epidemiology Program, Melbourne Sexual Health Centre, Alfred Health, Melbourne, VIC, Australia; j and Department of Epidemiology and Biostatistics, College of Public Health, Zhengzhou University, Zhengzhou, China

## Abstract

**Background:**

China has the highest prevalence of hepatitis B virus (HBV) infection worldwide. Universal HBV screening might enable China to reach the WHO 2030 target of 90% diagnostics, 80% treatment, and 65% HBV-related death reduction, and eventually elimination of viral hepatitis. We evaluated the cost-effectiveness of implementing universal HBV screening in China and identified optimal screening strategies.

**Methods:**

We used a Markov cohort model, inputting parameters based on data from previous studies and public databases, to assess the cost-effectiveness of four HBV serological screening strategies in China in different screening scenarios. We simulated universal screening scenarios in 15 adult age groups between 18 and 70 years, with different years of screening implementation (2021, 2026, and 2031) and compared to the status quo (ie, no universal screening); in total, we investigated 180 different screening scenarios. We calculated the incremental cost-effectiveness ratio (ICER) between the different screening strategies and the status quo (current screening strategy). We performed probabilistic and one-way deterministic sensitivity analyses to assess the robustness of our findings.

**Findings:**

With a willingness-to-pay level of three times the Chinese gross domestic product (GDP) per capita (US$30 828), all universal screening scenarios in 2021 were cost-effective compared with the status quo. The serum HBsAg/HBsAb/HBeAg/HBeAb/HBcAb (five-test) screening strategy in people aged 18–70 years was the most cost-effective strategy in 2021 (ICER $18 295/quality-adjusted life-years [QALY] gained). This strategy remained the most cost-effective, when the willingness-to-pay threshold was reduced to 2 times GDP per capita. The two-test strategy for people aged 18–70 years became more cost-effective at lower willingness-to-pay levels. The five-test strategy could prevent 3·46 million liver-related deaths in China over the lifetime of the cohort. It remained the most cost-effective strategy when implementation was delayed until 2026 (ICER $20 183/QALY) and 2031 (ICER $23 123/QALY). Screening young people (18–30 years) will no longer be cost-effective in delayed scenarios.

**Interpretation:**

The five-test universal screening strategy in people aged 18–70 years, implemented within the next 10 years, is the optimal HBV screening strategy for China. Other screening strategies could be cost-effective alternatives, if budget is limited in rural areas. Delaying strategy implementation reduces overall cost-effectiveness. Early screening initiation will aid global efforts in achieving viral hepatitis elimination.

**Funding:**

National Natural Science Foundation of China.

## Introduction

Hepatitis B virus (HBV) infection is a major global health issue, with 257 million chronically infected individuals and 887 000 HBV-related deaths in 2015.^1^ China has the largest population living with HBV, accounting for one-third of the world's infected population.^2^ Approximately 90 million people live with chronic hepatitis B infection in China, with an estimated 300 000 HBV-related deaths per year.^3^ With such a large HBV-infected population, China's efforts to achieve the WHO 2030 target of 90% diagnostic coverage and 80% treatment coverage among eligible individuals will have a considerable global impact on HBV prevention and control.^4^

The wide availability of birth-dose HBV vaccination to both urban and rural communities has drastically reduced HBsAg prevalence in children aged 5 years in China to 0·2% in 2016.^5^ With state-funded HBV vaccination programmes only starting in 2002,^6^ most of China's adult population remains unvaccinated, with the risk for HBV via mother-to-child transmission still possible in resource-limited regions.^7^, ^8^ Regular HBV screening might improve early diagnosis and treatment.^9^ The Chinese Government has invested more than 7 billion renminbi (about US$1·1 billion) since 2010 to promote HBV testing.^10^ Nevertheless, only around 19% of patients with chronic hepatitis B infection have been diagnosed as of 2016,^11^ because free HBV screening was limited to pregnant women and couples with premarital status.^10^

Universal screening has not been proposed or implemented for chronic HBV infection. Previously, the high cost of antiviral drugs was a major barrier for HBV screening because HBV treatment and care were not affordable after diagnosis.^12^ Since 2018, availability of low-cost generic medications, such as tenofovir and entecavir, has increased and China's medical insurance subsidisation policies have been reformed, leading to updates in the Chinese HBV guidelines for primary care to recommend HBV testing for all people aged 18 years and older.^13^ How to implement a universal screening programme, which is potentially very costly, and how it might affect the population in China, remains unclear.

**Research in context**

**Evidence before this study**

Hepatitis B virus (HBV) screening is recommended for people born in regions with a disease prevalence of 2% or higher, but it has yet to be universally implemented in many HBV-endemic countries. China accounts for one-third of the world's HBV-infected population but has diagnostic and treatment coverages below the WHO 2030 goals. Improvements in health-care infrastructure and the introduction of low-cost generics could allow for universal HBV screening in China to be cost-effective. We searched PubMed, Embase, and Web of Science between Jan 1, 2000, and Feb 28, 2021, with no language restrictions, using the terms “China” or “Chinese”, “Hepatitis B”, “HBV”, “screening”, “test”, “diagnosis”, and “cost-effectiveness” to identify published economic evaluations on universal HBV screening strategies. We found no previous studies describing the costs or cost-effectiveness of universal screening for HBV infection in mainland China. We also searched for studies on the cost-effectiveness of HBV screening in other countries using the same search terms, without “China” or ”Chinese”. Only three previous studies evaluated the cost-effectiveness of one type of HBV screening in high-income countries and focused on high-risk or immigrant populations. One study evaluated the cost-effectiveness of community-based screening in a low-income country, but it only considered the HBsAg rapid test for HBV screening.

**Added value of this study**

Based on our cost-effectiveness analysis of 180 screening scenarios, universal HBV screening is cost-effective and can be applied to every screening strategy if implemented early. The most cost-effective strategy was the serum HBsAg/HBsAb/HBeAg/HBeAb/HBcAb (five-test) screening strategy in people aged 18–70 years. This strategy remained robustly cost-effective even after reductions in willingness-to-pay levels. Different screening strategies were suitable for different willingness-to-pay thresholds, allowing for generalisation to the whole of China or individual application to different Chinese regions. Early initiation of universal HBV screening could potentially identify 86·8% of infected individuals by the end of the cohort's lifetime, and could potentially save 3·46 million Chinese individuals from HBV-related mortality. Although a delay in screening implementation by 5–10 years did not alter our conclusions, overall cost-effectiveness was reduced, with the exclusive screening of the younger age groups (18–30 years and 18–40 years) no longer being cost-effective.

**Implications of all the available evidence**

Universal HBV screening is cost-effective in China, especially when implemented early. Delay in screening implementation reduces cost-effectiveness. It is now prime time for the initiation of universal HBV screening, which will improve the current suboptimal diagnostic and treatment rates for HBV infection in China, and will help China to achieve the WHO 2030 objectives of eliminating HBV as a public health threat.

This study evaluates the effectiveness and cost-effectiveness of various universal HBV screening strategies for all Chinese adults compared with the current practice. We also conducted the simulation at different years of initiation at 2021, 2026, and 2031 to understand the effect of early or delayed implementation.

## Methods

### Study design

We conducted an economic evaluation based on a decision-analytic model to assess the cost-effectiveness of various HBV universal screening strategies in China. The model was constructed using TreeAge Pro 2020 and the analysis was reported according to the Consolidated Health Economic Evaluation Reporting Standards statement.^14^

### Data source

We collected data from a literature search and Chinese and English public databases to parameterise prevalence data, screening, cost, and other model parameters (appendix pp 1–3). Costs were collected from the health provider's perspective, consisting of the direct medical costs of screening, vaccines, and treatment for active chronic hepatitis B infection, compensated cirrhosis, decompensated cirrhosis, hepatocellular carcinoma, and liver transplantation. All costs were expressed in 2020 US dollars. The annual transition probabilities and utility scores were derived from published literature on the natural history and treatment progression of active chronic hepatitis B infection to hepatocellular carcinoma (appendix p 1). Background age-specific mortality was obtained from the China Population and Employment Statistics Yearbook, 2019.^15^

### Modelling

Mathematical models have been widely used to evaluate prevention programs for sexually transmitted infections.^16^, ^17^ A Markov model (appendix p 4) was constructed to simulate disease progression of HBV infection to cirrhosis and hepatocellular carcinoma in a cohort of 100 000 people aged 18–70 years, with a yearly time-step over a life expectancy of 80 years; its age structure resembled the age distribution of the Chinese population in 2020.^18^ The model consisted of 11 health states from disease susceptibility to hepatocellular carcinoma. We assumed that primary care providers would carry out screening for HBV and follow-up treatment. We assumed no transmission in the model, as HBV transmission in China was largely driven by mother-to-child transmission, which was low due to successful prevention programmes.^19^ In the absence of screening, unvaccinated individuals would be diagnosed only after developing clinical symptoms. A proportion of HBsAg-positive individuals would progress to active chronic hepatitis B infection, compensated cirrhosis, decompensated cirrhosis, hepatocellular carcinoma, and HBV-related death, or regress to the resolved infection state (appendix p 4). The initial age-specific distribution of HBV-positive patients was informed by infection prevalence from published studies (appendix p 5).

We assumed that health state transition rates differed between participants on treatment and those who remained untreated. We defined treatment-eligible individuals as HBsAg-positive individuals (diagnosed and undiagnosed) with a high viral load (≥2000 IU/mL), cirrhosis or hepatocellular carcinoma, or who have undergone liver transplantation according to the international and local guidelines.^13^ Treatment-eligible patients were prescribed to antiviral therapy. In the model, we applied age-adjusted vaccine rates based on published studies (appendix p 5).

Chinese age-specific natural death rates were applied to the cohorts.^15^ Additionally, people with active chronic hepatitis B infection, compensated cirrhosis, decompensated cirrhosis, hepatocellular carcinoma, and liver transplantation would face cause-specific mortality from these diseases. We evaluated the effectiveness and cost-effectiveness of screening strategies based on model outputs, including the overall investment cost, the number of HBV-infected individuals diagnosed and treated, the number of non-infected individuals vaccinated, and quality-adjusted life-years (QALYs) for each screening strategy.

### Screening strategies and scenarios

We defined the current practice of HBV screening in China as the status quo. Since 2017, only couples undergoing premarital testing, pregnant women, and individuals who developed clinical symptoms were eligible to receive screening.^10^

We simulated universal HBV screening to target age subgroups between 18–70 years in the Chinese population (life expectancy 77 years in 2020^20^). The frequency of screening was once per lifetime for all individuals in their respective age cohorts. We assumed that screening was implemented in the first year of cohort initiation. Based on the current diagnostic practice, we investigated four screening strategies: HBsAg rapid test;^21^ the combined HBsAg/HBsAb test (two-test); the combined HBsAg/HBsAb/HBcAb test (three-test); and the combined HBsAg/HBsAb/HBeAg/HBeAb/HBcAb test (five-test). Individuals with a positive rapid test would receive a confirmatory five-test. Results from the two-test, three-test, and five-test provided additional information for disease staging. Sensitivities and specificities of screening methods were estimated using Chinese-specific data.^22^, ^23^

Treatment-eligible patients would be offered liver ultrasonography, HBV DNA testing, or transient elastography to assess liver fibrosis. Standard treatment was conducted following international and local guidelines.^9^, ^24^

A previous study recommended that all HBV-seronegative individuals receive the HBV vaccine,^25^ and 55–72% acceptance rates were assumed if the vaccine was offered free of charge.^26^ We assumed lifetime protection of HBV vaccines.

We examined screening strategies in 15 target groups stratified by age, which included five age groups (18–30, 18–40, 18–50, 18–60, and 18–70 years) born before the HBV vaccine was integrated into the expanded Chinese national programme on immunisation and was made free in 2002.^27^ Four age groups (30–40, 30–50, 30–60, 30–70 years) were born before the availability of self-funded HBV immunisation in 1992. We constructed the scenarios with various initiating years (2021, 2026, and 2031) to estimate the effect of early or delayed universal screening. In total, our model investigated 180 screening scenarios.

### Cost-effectiveness analysis

We calculated an incremental cost-effectiveness ratio (ICER—ie, cost/QALY gained) between current practice (status quo) and the different screening strategies to determine the screening strategy's cost-effectiveness. We adopted the WHO definition of cost-effectiveness of fewer than three times Chinese gross domestic product (GDP) per capita.^28^ The willingness-to-pay threshold was set as three times Chinese GDP per capita based on the WHO-CHOICE and previous publications ($30 828 in 2019^29^, ^30^). Future costs and QALYs were discounted at 3% per year.^31^, ^32^

### Sensitivity analysis

We conducted probabilistic sensitivity analyses to characterise all model parameters’ combined uncertainty based on 1000 Monte Carlo simulations. We conducted one-way deterministic sensitivity analyses to determine the effects of parameter uncertainties and model robustness (tornado plots). We evaluated the impact of varying treatment coverage (10–80%) on the ICER, based on an about 30% coverage rate as in other east Asian countries^5^ and WHO 2030 targets of 80%.

### Role of the funding source

The funder of the study had no role in the study design, data collection, data analysis, data interpretation, or writing of the report.

## Results

If the status quo continued and no further scale-up of screening strategies was implemented, only 19·0% of the study population would be screened by the end of the cohort's lifetime. An estimated 6·4% (6440/100 000) would be living with HBV infection over their lifetime; 18·9% (1217/6440) of infected individuals would be diagnosed; and 12·6% (310/2460) of eligible HBV-infected individuals would receive treatment. 23·0% (1479/6440) of infected individuals would eventually die of HBV-related diseases. The overall screening cost would be $267 000 and treatment cost would be $13·6 million for the status quo over the lifetime of the 100 000-person cohort (table).

**Table:** Effectiveness and cost-effectiveness of various HBV universal screening strategies in a cohort of 100 000 Chinese individuals

	**Intervention effectiveness**						**Investment cost**			**Cost-effectiveness**	
	Population screened, %	Population vaccinated after screening*	Individuals living with HBV diagnosed over the lifetime	Eligible HBV-infected individuals who received treatment†	QALYs accumulated over the lifetime	HBV-related deaths over the lifetime (per 100 000)	Cost for screening, US$ thousand	Cost for vaccination, US$ thousand	Cost for HBV treatment, US$ thousand	Cost per death averted, US$	ICER cost/QALY gained
**Initiating screening in 2021**											
Status quo	19·0%‡	0·0%	18·9%	12·6%	1 465 674	1479	$267	$0	$13 603	..	..
Five-test, 18–70 years	76·2%	53·6%	86·8%	38·4%	1 467 225	1230	$1066	$253	$15 370	$15 107	18 295
Two-test, 18–70 years	76·2%	46·7%	85·9%	36·4%	1 467 197	1241	$633	$220	$15 317	$14 770	2783
Two-test, 18–60 years	65·4%	36·4%	74·1%	33·5%	1 466 996	1293	$404	$171	$15 038	$15 414	1950
Two-test, 18–50 years	53·5%	25·0%	59·2%	29·4%	1 466 700	1348	$385	$118	$14 533	$18 292	1351
Two-test, 18–40 years	41·4%	14·0%	51·2%	23·7%	1 466 327	1418	$298	$66	$14 167	$33 394	1063
Two-test, 18–30 years	30·8%	5·5%	43·6%	19·1%	1 465 979	1457	$283	$26	$13 853	$67 823	954
**Initiating screening in 2026**											
Status quo	19·0%‡	0·0%	18·9%	12·6%	1 467 408	1483	$258	$0	$12 397	..	..
Five-test, 18–70 years	76·4%	48·0%	85·4%	37·8%	1 468 796	1271	$916	$226	$14 201	$15 999	20 183
Three-test, 18–70 years	76·4%	45·3%	85·0%	37·5%	1 468 774	1280	$808	$214	$13 891	$15 185	19 551
Two-test, 18–70 years	76·4%	40·9%	84·5%	36·0%	1 468 770	1285	$572	$193	$14 058	$14 600	2795
Two-test, 18–60 years	63·8%	31·2%	71·5%	33·1%	1 468 572	1319	$383	$147	$13 740	$15 520	1934
Two-test, 18–50 years	53·0%	21·9%	54·4%	28·4%	1 468 283	1374	$364	$103	$13 244	$18 417	1344
Two-test, 18–40 years	40·6%	12·9%	46·1%	23·2%	1 467 930	1431	$293	$61	$12 883	$38 054	1222
Two-test, 30–40 years	29·3%	5·1%	37·4%	18·4%	1 467 746	1460	$281	$24	$12 707	$62 713	1056
**Initiating screening in 2031**											
Status quo	19·0%‡	0·0%	18·9%	12·6%	1 469 338	1488	$244	$0	$11 024	..	..
Five-test, 18–70 years	74·7%	40·6%	82·8%	37·4%	1 470 548	1299	$788	$192	$12 820	$15 696	23 123
Three-test, 18–70 years	74·7%	38·9%	82·4%	37·1%	1 470 529	1305	$678	$183	$12 511	$13 951	20 804
Two-test, 18–70 years	74·7%	35·3%	81·9%	35·1%	1 470 525	1307	$507	$166	$12 605	$13 662	2810
Two-test, 18–60 years	62·9%	26·3%	66·2%	32·3%	1 470 330	1360	$328	$124	$12 279	$16 227	1910
Two-test, 18–50 years	50·7%	17·8%	49·9%	25·8%	1 470 052	1408	$310	$84	$11 806	$33 260	1649
Two-test, 30–50 years	39·6%	10·2%	44·3%	18·3%	1 469 941	1436	$281	$48	$11 688	$43 474	1357
Two-test, 30–40 years	29·1%	3·6%	31·4%	14·8%	1 469 614	1467	$275	$17	$11 281	$72 601	1107

**Figure 1:**
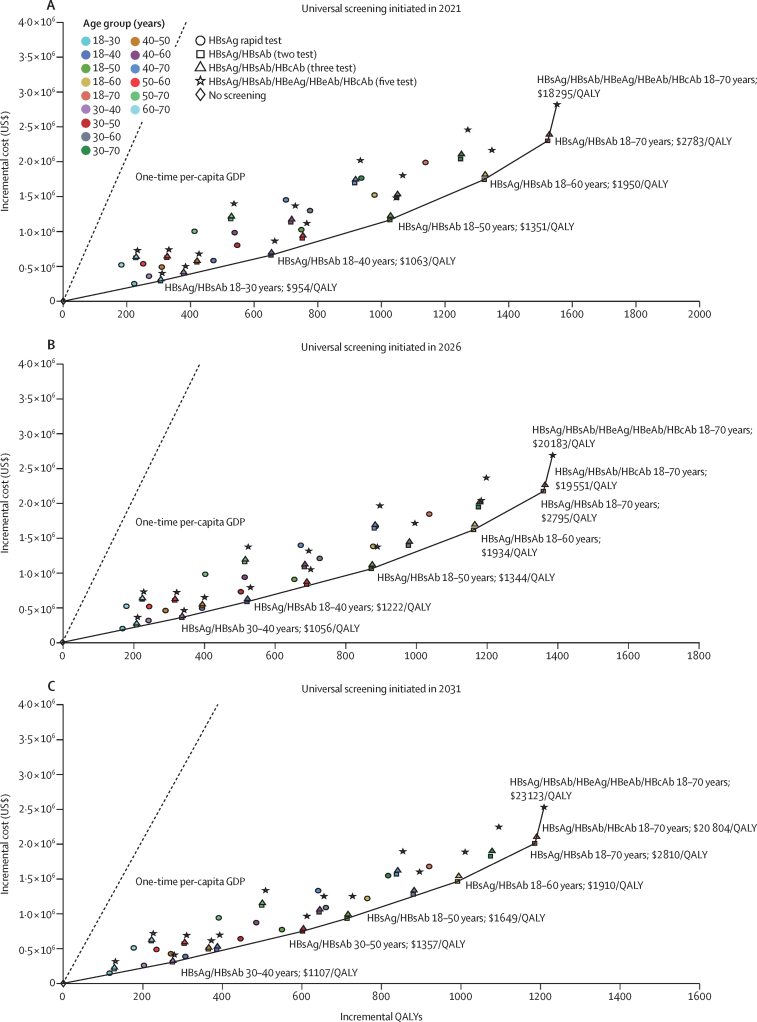
Cost-effectiveness planes for all HBV screening strategies by initiation year 2021 (A), 2026 (B), 2031 (C) Solid line=cost-effectiveness frontier. Strategies on the cost-effectiveness frontier dominate strategies above the frontier. ICER values were estimated by comparing the current screening strategy in China (status quo) with the next screening strategy on the frontier within the same year. GDP=gross domestic product. HBV=hepatitis B virus. ICER=incremental cost-effectiveness ratio. QALY=quality-adjusted life-years.

The age distribution of the cohort resembles the age distribution of the Chinese population in 2020. Only the screening strategies on the cost-effectiveness frontier from figure 1 were compared in the table. HBV=hepatitis B virus. ICER=incremental cost-effectiveness ratio. QALY=quality-adjusted life-year.

* Assuming all HBV-seronegative individuals will be referred for vaccination, with a 55–72% vaccine acceptance rate (appendix p 3).

† Treatment coverage rate was estimated from available published data (appendix p 3), with further adjustment based on HBV serology and virology data in China (appendix p 3).

‡ Proportion of the population screened via currently available methods (premarital and antenatal)—ie, the status quo.

The incremental costs and QALYs for the 60 universal HBV screening scenarios (ie, four screening methods in 15 cohorts, initiated in 2021) compared with the status quo is shown in the appendix (p 6). All screening strategies were non-dominated. The youngest age group (18–30 years) had the lowest cost per QALY gained ($954) compared with the status quo with the two-test strategy, while the cost was the highest ($3132) in older age group (60–70 years) with the five-test strategy (appendix p 6).

We identified six screening strategies on the cost-effectiveness frontier (figure 1A). The five-test strategy for the target group aged 18–70 years (ICER $18 295/QALY gained) was the most cost-effective strategy in 2021. This was followed by the two-test strategy for those aged 18–70 years ($2783/QALY gained), the two-test strategy for those aged 18–60 years ($1950/QALY gained), the two-test strategy for those aged 18–50 years ($1351/QALY gained), the two-test strategy for those aged 18–40 years ($1063/QALY gained), and the two-test strategy for those aged 18–30 years ($954/QALY gained). While rapid tests were cost-effective compared with the status quo, they were dominated by strategies on the frontier.

If the five-test strategy was initiated among the population aged 18–70 years in 2021, 86·8% of infected individuals would be diagnosed by the end of the cohort's lifetime, and 38·4% of treatment-eligible HBV-infected individuals would receive treatment (table). We projected that this screening strategy had the lowest deaths (1230 deaths per 1 000 000 population) among all the strategies, which means it had a reduction of 249 HBV-related deaths (reduced by 16·8% [249/1479]) per 100 000 population compared with the status quo (1479 deaths). Yet, when taking China's 1·39 billion people into consideration, this strategy would save 3·46 million Chinese individuals from HBV-related mortality.

If universal screening was initiated in 2026, screening strategies involving an upper age range of 40 years or older remained cost-effective but to a lesser extent (figure 1B). The five-test strategy for those aged 18–70 years remained the most cost-effective, although the ICER increased to $20 183/QALY gained. The next most cost-effective were the two-test strategy for those aged 18–70 years ($2795/QALY gained), and the two-test strategy for those aged 18–60 years ($1934/QALY gained), 18–50 years ($1344/QALY gained), 18–40 years ($1222/QALY gained), and 30–40 years ($1056/QALY gained). Screening individuals aged 18–30 years would be dominated by other strategies and would no longer be the most cost-effective strategy. 1271 deaths per 100 000 population were estimated with the five-test screening strategy for 18–70 years, initiated in 2026, compared with 1483 deaths with the status quo, so the five-test strategy would advert 212 more deaths over the lifetime of the 100 000-person cohort (table).

If universal screening was to be initiated in 2031, the cost-effectiveness would be further reduced (figure 1C). The five-test strategy for those aged 18–70 years remained the most cost-effective (ICER increased to $2323/QALY gained). The three-test strategy for ages 18–70 years had an ICER of $20 804/QALY gained. The two-test strategy for 18–70 years had an ICER of $2810/QALY gained, for 18–60 years it was $1910/QALY gained, for 18–50 years it was $1649/QALY gained, for 30–50 years it was $1357/QALY gained, and for 30–40 years it was $1107/QALY gained. Screening individuals aged 18–30 years and 18–40 years would no longer be cost-effective with any strategy if implemented in 2031. The five-test strategy for 18–70 years was projected to have 1299 deaths per 100 000 population, which would avert 189 more deaths over the lifetime of the 100 000-person cohort compared with the status quo (1488 deaths; table).

**Figure 2:**
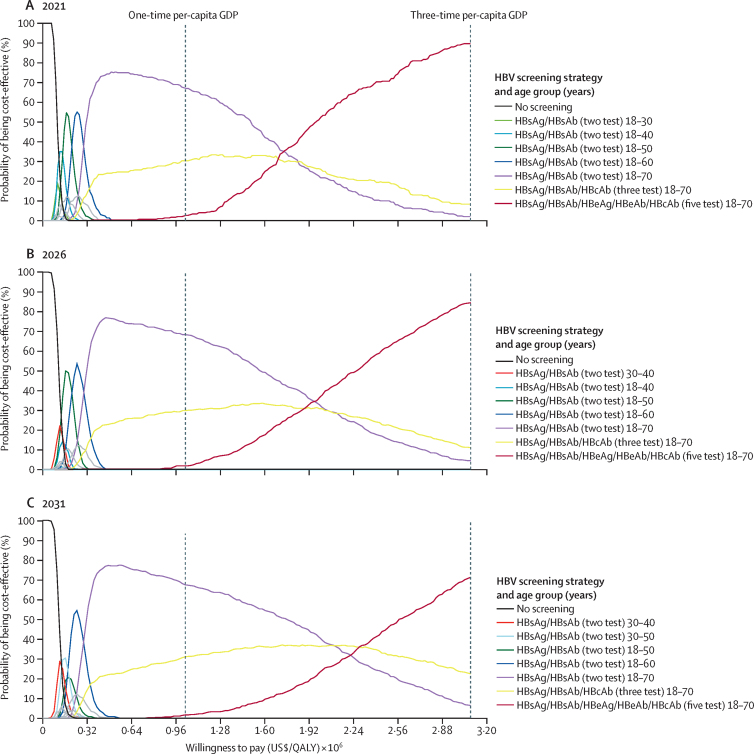
Cost-effectiveness acceptability curves for all HBV universal screening strategies by initiation year 2021 (A), 2026 (B), and 2031 (C) GDP=gross domestic product. HBV=hepatitis B virus. QALY=quality-adjusted life-years.

The probability of a strategy being cost-effective varied depending on the willingness-to-pay threshold (0–3 times GDP per capita; figure 2). At a threshold of $30 828 (3 times GDP per capita), the five-test strategy initiated in 2021 for those aged 18–70 years had an 89·6% probability of being cost-effective, outperforming other strategies initiated in that year (figure 2A). The five-test strategy at 18–70 years remained the leading cost-effective strategy until 1·73 times the GDP per capita ($17 778), after which point the two-test strategy for 18–70 years became the most cost-effective (68·3% at one-time GDP per capita). If willingness-to-pay was decreased to 30% GDP per-capita, the two-test strategy for 18–60 years and two-test strategy for 18–50 years would be the most cost-effective. If screening initiation was delayed until 2026 and 2031, the five-test strategy for those aged 18–70 years had a lower probability of being the most cost-effective (84·7%, and 71·2%, respectively) at 3 times GDP per capita than if implemented in 2021. It only retained the highest probability of cost-effectiveness when willingness-to-pay was above 1·97 times GDP per capita ($20 244) for 2026 and 2·29 times GDP per capita ($23 532) for 2031 (figures 2B, C).

Variations in all parameters did not substantially affect the conclusion that the five-test strategy for individuals aged 18–70 years was the most cost-effective screening strategy for the Chinese population (appendix p 7). The cost of the five-test strategy had the greatest effect on ICER: if the screening costs were reduced to the lowest end of the cost range ($10·5), then the ICER would be reduced to $10 600; in contrast, if this cost increased to the upper end ($17), the ICER would increase to $26 534.

## Discussion

International guidelines recommend HBV screening for people born in regions with 2% or higher disease prevalence.^24^, ^33^ Yet, universal screening has not been implemented in most HBV-endemic countries because of concerns related to high costs and insufficient infrastructure for implementation. Our study shows the cost-effectiveness of universal HBV screening in China, with the most cost-effective strategy being to screen 18–70-year-olds with a five-test screening strategy. Delaying screening by 5–10 years did not change this conclusion but its cost-effectiveness was reduced, with certain populations’ screening being no longer cost-effective by 2031. The five-test sampling, recommended by Chinese guidelines^34^ was the most commonly used strategy in China, especially in the hospital setting, while the rapid test might be more accessible in rural settings**.** However, only in the case of a very low budget for screening (willingness-to-pay of 30% GDP per-capita) did we find that low-cost rapid tests for all age groups would be the most cost-effective option. Furthermore, we recommend prioritising screening to the youngest age group (18–30 years) before extending the programme to the older age groups, if additional resources become available. To our knowledge, this is the first economic evaluation study of universal screening and treatment strategy for HBV in China.

Our study has several important findings. First, universal HBV screening in China is cost-effective regardless of screening strategies if implemented in 2021. The five-test strategy is generally the most cost-effective option. Our sensitivity analysis (appendix p 7) illustrated that the cost of the five-test strategy is the most influential parameter affecting cost-effectiveness. It has a marginal cost increase of $8·55 per test compared with the two-test strategy (appendix p 7), yet it provides a comprehensive HBV serological profile, facilitating disease staging and linkage-to-care. While the rapid point-of-care test remains a cost-effective choice compared with the status quo, the extended dominance (ie, higher incremental cost-effectiveness ratio compared with other effective strategies) means it is a less preferable option, due to its suboptimal sensitivity and the need for confirmatory testing.^21^ Notably, the youngest age group (18–30 years) would benefit most from screening because identifying younger people who need vaccination would result in greater QALYs gained. Vaccinating young people, who will have a longer living lifespan, will result in more QALYs from a fixed investment sum. Figure 1A can be interpreted as an expansion path for various-sized budgets. Also, as age increases, the ICERs become less favourable because the lifespan of the ageing population would be reduced, regardless of subsequent treatment, when compared with the younger age group (appendix p 6).

Second, our study findings are robust and can be generalised to China as a nation or applied individually to smaller regions and provinces. A health-care intervention is deemed cost-effective if the ICER is below the willingness-to-pay threshold.^28^ However, these thresholds and their use are contested, especially in low-income and middle-income countries where various thresholds have been suggested. This contention is especially relevant in China, where variations in GDP per capita are substantial across Chinese regions and provinces. Hence, we conducted probabilistic sensitivity analyses, which confirms the five-test strategy for 18–70 years in 2021 as the most cost-effective strategy, and would remain as such even if the willingness-to-pay threshold was lowered to 2 times GDP per capita (figure 2A). We further demonstrated that, at a lower willingness-to-pay threshold (<one time GDP per capita), a less expensive strategy, such as the two-test strategy, would be a viable option in rural China. Additionally, as the disease burden of HBV varies substantially across various regions in China,^35^ we suggest universal screening to be initiated with priority in provinces with higher prevalence.

Third, our analysis suggests that a delay of 5–10 years in screening implementation will reduce its cost-effectiveness (figures 1B, C), with exclusive HBV screening in the younger age groups (18–30 years or 18–40 years) no longer being cost-effective. The findings align with the projection that vaccinated newborns (most being born after 2002) will mature into early-adulthood and mid-adulthood over the next decade, which implies that if the initiation of screening is further delayed to 2026 or 2031, the QALYs gained by an increasing proportion of people who are already vaccinated would be less than the QALYs gained by a decreasing proportion of people who are unvaccinated. Although the five-test strategy for 18–70 years remains the most cost-effective strategy regardless of the timing of strategy initiation, the cost-effectiveness has been reduced because the ageing population infected with HBV are dying of HBV-related diseases in the delayed period, which would decrease the beneficial effects of screening. Therefore, initiating universal HBV screening as soon as possible represents the best opportunity to maximise the population benefits and avoid deaths, especially when an early diagnosis of HBV at a younger age substantially reduces the risk of future liver-related complications.^36^ Moreover, previous studies have demonstrated the effects of HBV screening on improving long-term survival rates.^37^ From a practical perspective, planning universal screening will require time; however, our study confirms that initiating HBV universal screening over the coming decade remains a very viable option from an economic perspective.

Even with universal screening involving adults aged up to 70 years, the decline in HBV-related mortality is only relatively modest at 16·8% compared with the status quo in 2021. The study findings are similar to those of a previous modelling study using a case-finding approach,^38^ indicating that the WHO 2030 target of a 65% reduction in mortality might be unrealistic for China. 249 deaths per 100 000 corresponds to 3·46 million deaths in a population of 1·39 billion. Other health-care interventions, including liver cancer surveillance and improving the care continuum^39^ will be needed to reduce mortality further. That said, universal screening is associated with downstream improvements in linkage-to-care rates.^40^ A universal HBV screening programme in The Gambia achieved an 81% success in subsequent linkage-to-care,^40^ suggesting widespread screening could also increase treatment coverage.

Although our study findings demonstrate the clinical and economic benefits and the urgency of universal HBV screening in China, it does have limitations. It does not consider the real-world practicality of mass screening using China's current health-care infrastructure. The Chinese Government should make use of the extensive primary care infrastructure introduced since health-care reform in 2009. Our nationally representative survey among 149 community health centres and 3580 frontline primary care practitioners from 20 cities, found 80% of community health centres had the facility to offer HBV testing, and 85% of doctors saw the benefits of providing HBV testing, yet only 19% had diagnosed HBV and 15% managed HBV in the previous month.^41^ Insufficient training (54% of respondents) and financial support (23%) were cited as the major barriers to not offering HBV care. With health financing reform and the percentage of out-of-pocket health expenditure at the lowest point in 20 years,^42^ China's health-care infrastructure might be at its most receptive state to oversee the logistical and administrative demands of a national HBV screening programme. Additionally, the COVID-19 pandemic might have short-term and long-term positive and negative effects on primary health care, and these were not included in the model. Another limitation was that available medical literature used to construct utility scores for our cost-effectiveness analysis was mainly from specialised tertiary centres and might not represent Chinese society as a whole. To compensate for this limitation, we included studies published in simplified Chinese and data originating from China's more rural areas. Finally, as a decision-tree model, all projections were based on the current status, and as we cannot predict the future change of the relevant health policies, we did not consider the impacts of policy updates on HBV diagnosis and treatment in our model.

In conclusion, universal screening for HBV among adults aged 18–70 years is cost-effective in China, with various strategies suitable for various willingness-to-pay thresholds. A universal screening programme will still be cost-effective even with a 5–10 year delay, although reduced. Initiating universal HBV screening as soon as possible is recommendable for HBV prevention in China. It might improve the current suboptimal rates of diagnostic and treatment coverage and help China achieve the WHO objective of eliminating HBV as a public health threat.

## Data sharing

All data relevant to the study are included in the Article or in the online appendix.

## Declaration of interests

PC is a staff member of WHO; PC alone is responsible for the views expressed in this publication, and they do not necessarily represent the decisions or policies of WHO. M-FY is an advisory board member or has received research funding from AbbVie, Arbutus Biopharma, Assembly Biosciences, Bristol Myers Squibb, Dicerna Pharmaceuticals, GlaxoSmithKline, Gilead Sciences, Janssen, Merck Sharp and Dohme, ClearB Therapeutics, and Springbank Pharmaceuticals; and has received research funding from Arrowhead Pharmaceuticals, Fujirebio Incorporation, and Sysmex Corporation. W-KS received speaker's fees from AstraZeneca and Mylan, is an advisory board member of CSL Behring, is an advisory board member and received speaker's fees from AbbVie, and is an advisory board member and received speaker's fees and researching funding from Gilead Sciences. FJ received speaker's fees from Gilead Sciences, Merck Sharp and Dohme, and Bristol Myer Squibb, and is an advisory board member of Gilead Sciences and Merck Sharp and Dohme. All remaining authors declare no competing interests.
