# Supplementary material for: Cost-effectiveness of universal screening for chronic hepatitis B virus infection in China: an economic evaluation
Source: Lancet Glob Health. 2022 Jan 18;10(2):e278–87. doi: 10.1016/S2214-109X(21)00517-9 (PMC8789560; doi:10.1016/S2214-109X(21)00517-9)
Supplement: Supplementary appendix [file mmc1.pdf]

THE LANCET
Global Health

Supplementary appendix

This appendix formed part of the original submission and has been peer reviewed. We post it as supplied by the authors.

Supplement to: Su S, Wong WCW, Zou Z, et al. Cost-effectiveness of universal screening for chronic hepatitis B virus infection in China: an economic evaluation. *Lancet Glob Health* 2022; **10:** e278–87.

Appendix:
Table S1. A list of parameters used in the decision-analytic Markov model.

	Base case	Range	PSA distribution	Data source
Natural disease progression				
From chronic HBV carrier to:				
Active CHB	Age 20-39: 0.0023<br>Age ≥40: 0.0054	Age 20-39: 0.00115-0.00345<br>Age ≥40: 0.0027-0.0081	Age 20-39: Beta (3.99, 1730.14)<br>Age ≥40: Beta (3.97, 731.77)	[1-5]
From active CHB to:				
Compensated cirrhosis	0.029	0.015-0.058	Beta (4.14, 138.53)	[6]
Hepatocellular carcinoma	Age 20-39: 0.002<br>Age ≥40: 0.0061	Age 20-39: 0.001-0.003<br>Age ≥40: 0.00305-0.00915	Age 20-39: Beta (3.99, 1991.01)<br>Age ≥40: Beta (3.97, 657.7)	[2,3,5,7,8]
From Compensated cirrhosis to:				
Decompensated cirrhosis	0.073	0.03-0.1	Beta (5.42, 68.77)	[9-14]
Hepatocellular carcinoma	0.034	0.01-0.1	Beta (2.76, 78.35)	[7,11,14-18]
HBV-related death	0.031	0.031–0.038	Beta (9.28, 290.11)	[7,19]
From Decompensated cirrhosis to:				
Hepatocellular carcinoma	0.034	0.01-0.1	Beta (1.90, 54.12)	[7,11,14-18]
HBV-related death	0.17	0.1-0.25	Beta (4.73, 23.07)	[7,14,20-22]
From Hepatocellular carcinoma to:				
HBV-related death	0.45	0.22-0.7	Beta (1.66, 2.02)	[11,15,22,23]
Treatment-related annual transition estimates				
From active CHB to:				
Compensated cirrhosis	0.002	0.001-0.002	Beta (3.99, 1991.01)	[24-27]
Hepatocellular carcinoma	0.002	0.001-0.002	Beta (3.99, 1991.01)	[28]
From Compensated cirrhosis to:				
Decompensated cirrhosis	0.019	0.009-0.046	Beta (3.52, 181.17)	[24,29]
Hepatocellular carcinoma	0.02	0.016-0.044	Beta (24.48, 1199.52)	[30,31]
HBV-related death	0.017	0.012-0.048	Beta (2.82, 163.29)	[32-35]
From Decompensated cirrhosis to:				
Hepatocellular carcinoma	0.024	0.006-0.081	Beta (2,47, 100.63)	[28,30,36]
Liver transplantation	0.017	0.001-0.042	Beta (1.25, 72.03)	[24,37]
HBV-related death	0.095	0.056-0.14	Beta (5.27, 50.25)	[32]
From Hepatocellular carcinoma to:				
Liver transplantation	0.006	0.001-0.03	Beta (1.43, 236.13)	[24,37]
HBV-related death	0.26	0.25-0.27	Beta (4.74, 13.50)	[38]
Utility weight				
Chronic HBV infection	0.79	0.74-0.84	Beta (51.63, 13.73)	[39-47]
Compensated cirrhosis	0.73	0.69-0.78	Beta (89.19, 32.99)	
Decompensated cirrhosis	0.65	0.63-0.67	Beta (369.04, 198.71)	

Hepatocellular carcinoma	0.38	0.36-0.41	Beta (223.44, 364.56)	
Liver transplantation	0.67	0.64-0.69	Beta (163.93, 80.74)	
Costs ($USD, 2020)				
Screening cost				
HBsAg rapid test	2.85	2.3-3.4	Gamma (26.85,9.42)	
HBsAg and HBsAb test ("Two tests")	5.7	4.5-7.0	Gamma (22.56,3.96)	
HBsAg/HBsAb/HBcAb ("Three tests")	8.55	6.8-10.3	Gamma (18.28,2.14)	
HBsAg/HBsAb/HBeAg/HBeAb /HBcAb ("Five tests")	14.25	10.5-17.0	Gamma (14.44,1.01)	
Test cost				
Liver function test	3.3	2.6-4	Gamma (22.22,6.73)	
HBV DNA test	12.8	10.2-15.4	Gamma (24.24,1.89)	
Transient elastography	21.2	17.0-25.4	Gamma (25.48,1.20)	
Vaccination cost				
Vaccination for adults (per item)	4.71	2.15-7.30	Gamma (3.28,0.70)	48
Treatment cost				
Generic entecavir 0.5mg * 21 tablets	0.55	0.44-1.66	Gamma(25.00,45.45)	49
Generic tenofovir# 300mg * 30 tablets	1.25	1-1.5	Gamma(25.00,20.00)	49
Branded entecavir 0.5mg * 7tablets	20.22	16.17-24.26	Gamma(24.93,1.23)	49
Branded tenofovir# 300mg * 30tablets	47.06	37.65-56.47	Gamma(25.01,0.53)	49
Active chronic hepatitis B	2760	2200-3310	Gamma (24.29,0.0088)	50
Compensated cirrhosis	4330	3460-5200	Gamma (24.77,0.0057)	50
Decompensated cirrhosis	5523	4420-6630	Gamma (25.21,0.0046)	50
Hepatocellular carcinoma	7301	5840-8760	Gamma (23.69,0.0032)	50
Liver transplantation	35701	28560-42840	Gamma (27.11,1.08)	50,51
After liver transplantation follow-up (per year)	8393	6710-10070	Gamma (24.37,0.0029)	52
Screening Methods				
Rapid HBsAg test				53
Sensitivity	0.9	0.891-0.908	Point-estimate	
Specificity	0.995	0.994-0.995	Point-estimate	
HbsAg/HbsAb ("Two tests")				54
Sensitivity	0.932	0.851-0.985	Point-estimate	
Specificity	0.931	0.851-0.999	Point-estimate	
HbsAg/HbsAb/HBcAb ("Three tests")				

Sensitivity	0.941	0.87-0.99	Point-estimate	
Specificity	0.98	0.922-0.999	Point-estimate	
HBsAg/HBsAb/HBeAg/HBeAb/HBcAb ("Five tests")				54
Sensitivity	0.955	0.889-0.994	Point-estimate	
Specificity	0.998	0.993-1.00	Point-estimate	
Discount rate: costs (%)	3	0-6	Point-estimate	55,56
Discount rate: health outcomes (%)	3	0-6	Point-estimate	55,56

PSA, probabilistic sensitivity analysis; CHB, chronic hepatitis B; HBsAg, hepatitis B surface antigen; HBsAb, hepatitis B surface antibody; HBeAg, **hepatitis B e antigen; HBeAb, hepatitis B e antibody; HBcAb,** hepatitis B core antibody.

#The widely-available form of tenofovir in China was tenofovir disoproxil fumarate. Tenofovir alafenamide only became available in China in late 2020 and was not included in this analysis.

<u>Citations for the table in the main text</u>

Assuming all HBV seronegative individuals will be referred for vaccination, with a 55-72% vaccine acceptance rate. [57]

Treatment coverage rate was estimated from available published data [58] with further adjustment based on HBV serology and virology data in China. [59-68]

Figure S1. Schematic diagram of the Markov model for HBV infection and disease progression (CHB, chronic hepatitis B; HBV, hepatitis B virus; HCC, hepatocellular carcinoma.)**.**

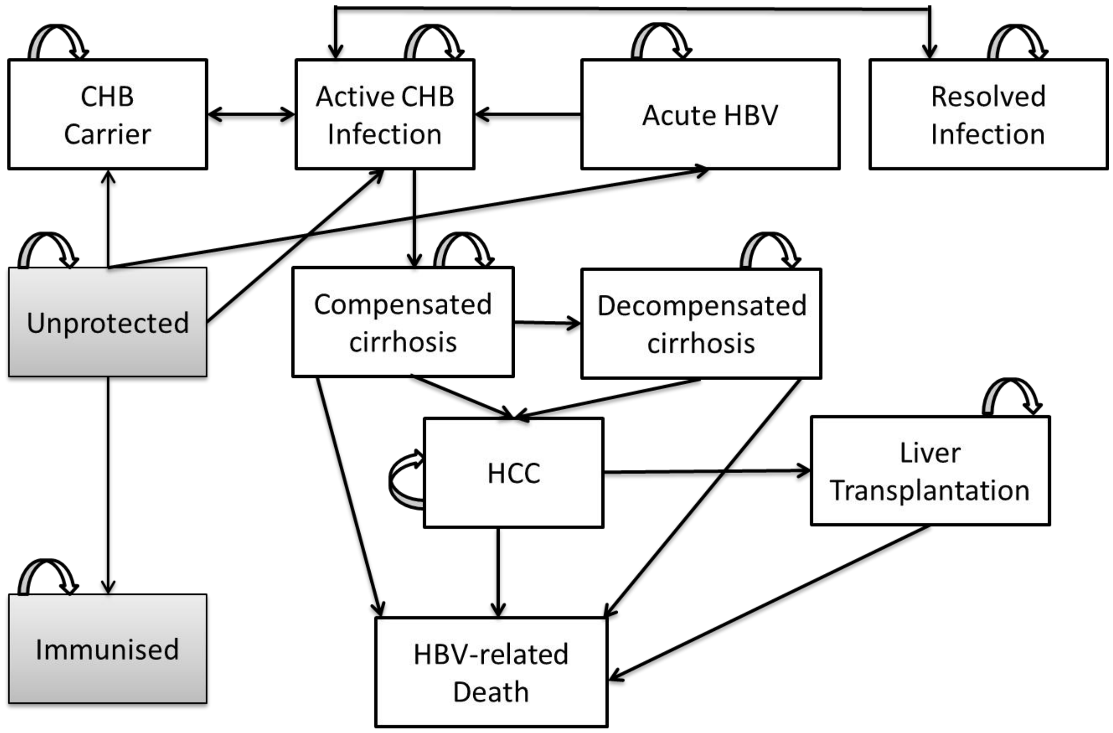

Figure S2. HBsAg+ rates and percentage of individuals who have received an HBV vaccine by age. Data were collected from published literature [59-64,66,67,69-84]. **Each coloured line represents an individual study** (HBsAg, hepatitis B surface antigen; HBV, hepatitis B virus)**.**

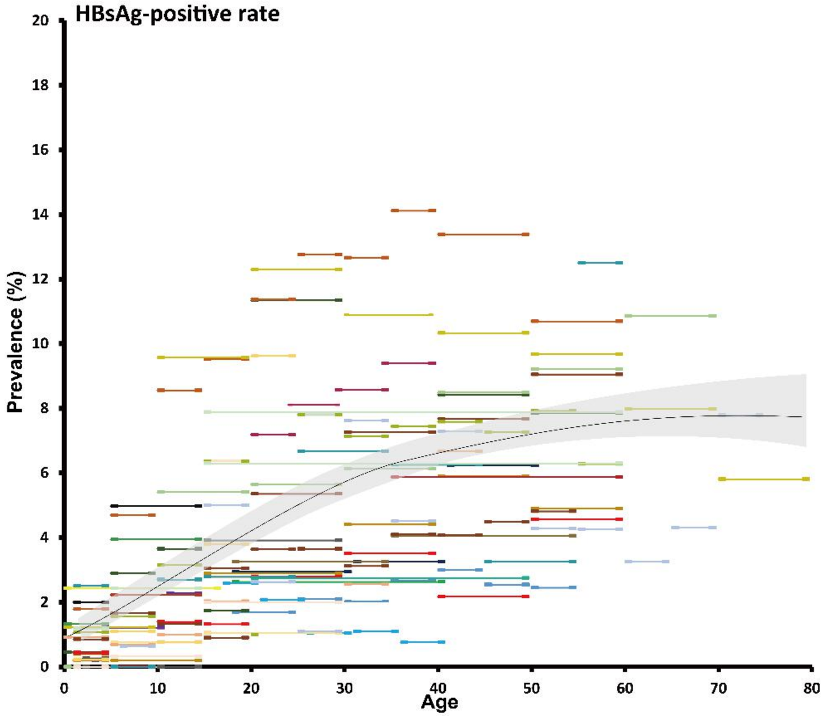

S2a

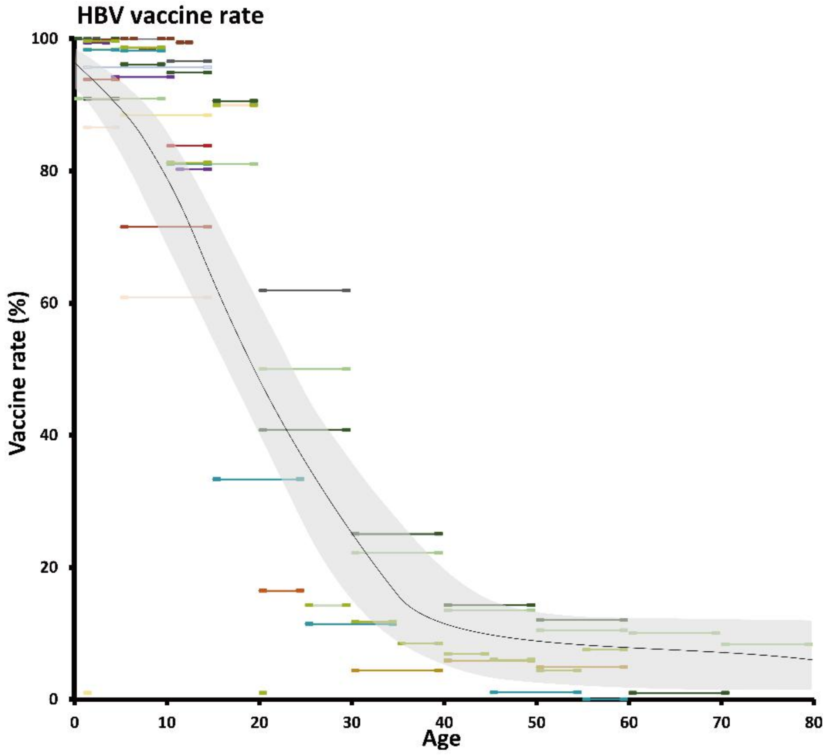

S2b

Table S2. Comparison of the incremental cost, incremental quality-adjusted life years (QALYs) and incremental cost-effectiveness ratio (ICER) of 60 HBV universal screening strategy initiated in 2021 with the status quo (19% background screening).

Screening age (Years)	Strategy screening method	QALYs	Incremental QALYs	Cost(US$)	Incremental costs (US$)	ICER vs. status quo* (US$/QALY)	ICER vs. next screening# (US$/QALY)
0-80	Status quo (19% background screening)	1,465,674		13,869,850			
18-70	HBsAg rapid test	1,466,813	1,139	15,856,574	1,986,724	1,744	1,744
	HBsAg/HBsAb	1,467,197	1,523	16,170,394	2,300,544	1,510	817
	HBsAg/HBsAb/HBcAb	1,467,201	1,528	16,257,855	2,388,005	1,563	19,590
	HBsAg/HBsAb/HBeAg/HBeAb/HBcAb	1,467,225	1,551	16,689,450	2,819,600	1,817	18,053
18-60	HBsAg rapid test	1,466,651	978	15,389,451	1,519,600	1,554	1,554
	HBsAg/HBsAb	1,466,996	1,323	15,612,544	1,742,694	1,318	647
	HBsAg/HBsAb/HBcAb	1,467,000	1,326	15,685,550	1,815,700	1,369	20,682
	HBsAg/HBsAb/HBeAg/HBeAb/HBcAb	1,467,021	1,347	16,034,875	2,165,025	1,607	16,798
18-50	HBsAg rapid test	1,466,422	749	14,896,090	1,026,240	1,370	1,370
	HBsAg/HBsAb	1,466,700	1,027	15,035,535	1,165,685	1,135	502
	HBsAg/HBsAb/HBcAb	1,466,703	1,029	15,091,979	1,222,129	1,187	23,294
	HBsAg/HBsAb/HBeAg/HBeAb/HBcAb	1,466,719	1,045	15,350,632	1,480,782	1,417	15,990
18-40	HBsAg rapid test	1,466,145	472	14,452,820	582,970	1,235	1,235
	HBsAg/HBsAb	1,466,327	653	14,530,708	660,858	1,012	430
	HBsAg/HBsAb/HBcAb	1,466,328	654	14,569,259	699,408	1,069	27,413
	HBsAg/HBsAb/HBeAg/HBeAb/HBcAb	1,466,338	665	14,736,251	866,401	1,303	16,211
18-30	HBsAg rapid test	1,465,897	224	14,121,467	251,617	1,125	1,125
	HBsAg/HBsAb	1,465,979	306	14,161,510	291,660	954	487
	HBsAg/HBsAb/HBcAb	1,465,980	307	14,183,229	313,378	1,022	29,362
	HBsAg/HBsAb/HBeAg/HBeAb/HBcAb	1,465,985	311	14,271,205	401,355	1,289	18,266
30-70	HBsAg rapid test	1,466,611	937	15,632,568	1,762,718	1,881	1,881
	HBsAg/HBsAb	1,466,922	1,248	15,909,208	2,039,358	1,634	890
	HBsAg/HBsAb/HBcAb	1,466,926	1,252	15,976,547	2,106,696	1,683	17,835
	HBsAg/HBsAb/HBeAg/HBeAb/HBcAb	1,466,945	1,272	16,327,244	2,457,394	1,933	17,910
30-60	HBsAg rapid test	1,466,450	776	15,165,445	1,295,595	1,669	1,669
	HBsAg/HBsAb	1,466,721	1,048	15,351,358	1,481,508	1,414	684
	HBsAg/HBsAb/HBcAb	1,466,724	1,051	15,404,241	1,534,391	1,460	18,614
	HBsAg/HBsAb/HBeAg/HBeAb/HBcAb	1,466,741	1,067	15,672,669	1,802,818	1,689	16,298
30-50	HBsAg rapid test	1,466,221	547	14,672,084	802,234	1,466	1,466
	HBsAg/HBsAb	1,466,425	752	14,774,350	904,500	1,203	499
	HBsAg/HBsAb/HBcAb	1,466,427	754	14,810,670	940,820	1,248	20,944
	HBsAg/HBsAb/HBeAg/HBeAb/HBcAb	1,466,439	766	14,988,425	1,118,575	1,461	15,000
30-40	HBsAg rapid test	1,465,944	270	14,228,814	358,964	1,329	1,329
	HBsAg/HBsAb	1,466,052	378	14,269,523	399,672	1,057	377
	HBsAg/HBsAb/HBcAb	1,466,052	379	14,287,950	418,100	1,104	25,688
	HBsAg/HBsAb/HBeAg/HBeAb/HBcAb	1,466,058	385	14,374,045	504,194	1,310	14,409
40-70	HBsAg rapid test	1,466,374	700	15,322,416	1,452,565	2,074	2,074
	HBsAg/HBsAb	1,466,590	916	15,564,234	1,694,384	1,850	1,122
	HBsAg/HBsAb/HBcAb	1,466,593	919	15,615,312	1,745,462	1,899	16,160
	HBsAg/HBsAb/HBeAg/HBeAb/HBcAb	1,466,607	933	15,890,506	2,020,655	2,165	19,203
40-60	HBsAg rapid test	1,466,213	539	14,855,292	985,442	1,827	1,827
	HBsAg/HBsAb	1,466,389	716	15,006,384	1,136,533	1,588	857
	HBsAg/HBsAb/HBcAb	1,466,391	718	15,043,007	1,173,157	1,634	16,451
	HBsAg/HBsAb/HBeAg/HBeAb/HBcAb	1,466,403	729	15,235,931	1,366,080	1,874	17,196
40-50	HBsAg rapid test	1,465,984	310	14,361,932	492,081	1,586	1,586
	HBsAg/HBsAb	1,466,093	420	14,429,375	559,525	1,333	616
	HBsAg/HBsAb/HBcAb	1,466,094	421	14,449,436	579,586	1,377	17,922
	HBsAg/HBsAb/HBeAg/HBeAb/HBcAb	1,466,101	427	14,551,687	681,837	1,595	15,495
50-70	HBsAg rapid test	1,466,086	413	14,872,352	1,002,502	2,428	2,428
	HBsAg/HBsAb	1,466,200	526	15,053,009	1,183,159	2,248	1,592
	HBsAg/HBsAb/HBcAb	1,466,202	528	15,085,518	1,215,668	2,301	15,209
	HBsAg/HBsAb/HBeAg/HBeAb/HBcAb	1,466,210	537	15,266,399	1,396,548	2,603	22,056
50-60	HBsAg rapid test	1,465,925	252	14,405,229	535,379	2,127	2,127
	HBsAg/HBsAb	1,465,999	326	14,495,159	625,309	1,919	1,213
	HBsAg/HBsAb/HBcAb	1,466,001	327	14,513,213	643,363	1,967	15,008
	HBsAg/HBsAb/HBeAg/HBeAb/HBcAb	1,466,006	332	14,611,823	741,973	2,234	19,374
60-70	HBsAg rapid test	1,465,857	183	14,391,557	521,706	2,844	2,844
	HBsAg/HBsAb	1,465,902	229	14,492,268	622,418	2,721	2,223
	HBsAg/HBsAb/HBcAb	1,465,903	230	14,508,487	638,636	2,780	15,409
	HBsAg/HBsAb/HBeAg/HBeAb/HBcAb	1,465,907	233	14,600,597	730,747	3,132	25,935

Willingness-to-pay (WTP), US$30,828;

* Screening strategies compared with the status quo (19% background screening);

# Screening strategy compared with next screening strategy within age group, e.g. HBsAg rapid test vs. status quo; HBsAg/HBsAb test vs. HBsAg rapid test; HBsAg/HBsAb/HBcAb test vs. HBsAg/HBsAb test; HBsAg/HBsAb/HBeAg/HBeAb/HBcAb test vs. HBsAg/HBsAb/HBcAb test;

Abbreviation: HBsAg, hepatitis B surface antigen; HBsAb, hepatitis B surface antibody; HBeAg, hepatitis B e antigen; HBeAb, hepatitis B e antibody; HBcAb, hepatitis B core antibody.

Figure S3. Tornado diagram demonstrating results of one-way sensitivity analyses of ten most important parameters that affect the cost-effectiveness of HBV universal screening with 'five-test' for 18-70 years.

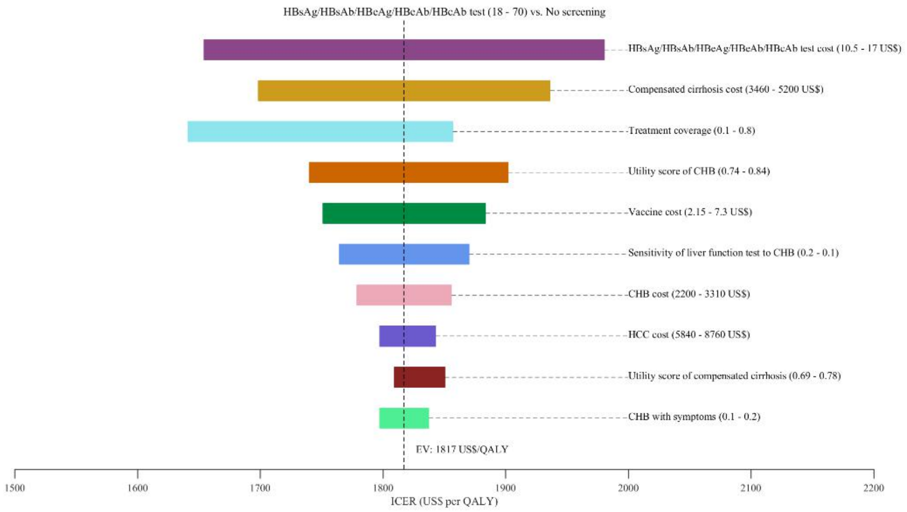

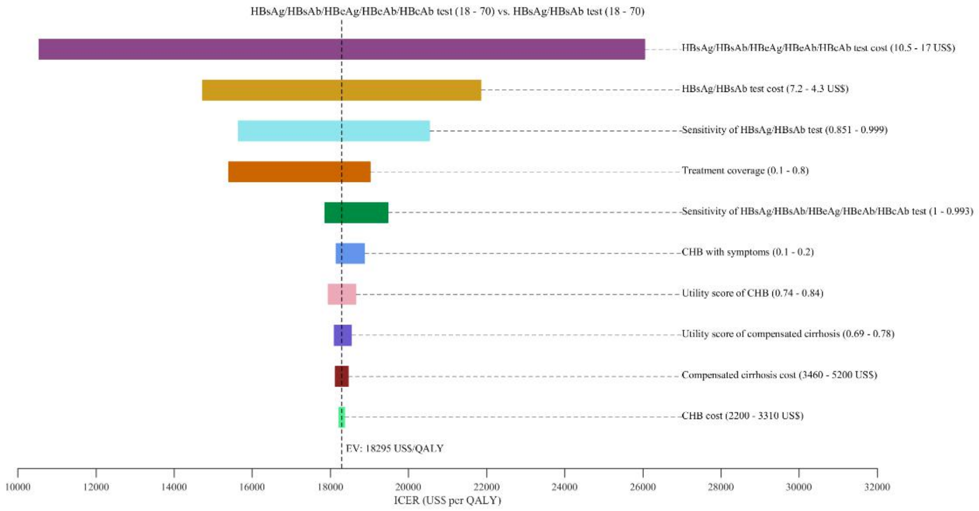

Abbreviation: CHB, chronic hepatitis B; HCC, hepatocellular carcinoma; ICER, incremental cost-effectiveness ratio; QALY, quality-adjusted life-years; HBsAg, hepatitis B surface antigen; HBsAb, hepatitis B surface antibody; HBeAg, hepatitis B e antigen; HBeAb, hepatitis B e antibody; HBcAb, hepatitis B core antibody.

Reference:

1. Hung HF, Chen TH. Probabilistic cost-effectiveness analysis of the long-term effect of universal hepatitis B vaccination: an experience from Taiwan with high hepatitis B virus infection and Hepatitis B e Antigen positive prevalence. *Vaccine* 2009; **27**(48): 6770-6.
2. Jacobs RJ, Saab S, Meyerhoff AS. The cost effectiveness of hepatitis immunization for US college students. *Journal of American college health : J of ACH* 2003; **51**(6): 227-36.
3. Tilson L, Thornton L, O'Flanagan D, Johnson H, Barry M. Cost effectiveness of hepatitis B vaccination strategies in Ireland: an economic evaluation. *European journal of public health* 2008; **18**(3): 275-82.
4. La Torre G, Mannocci A, Saulle R, et al. Economic evaluation of HBV vaccination: A systematic review of recent publications (2000-2013). *Human vaccines & immunotherapeutics* 2016; **12**(9): 2299-311.
5. Fendrick AM, Lee JH, LaBarge C, Glick HA. Clinical and economic impact of a combination Haemophilus influenzae and Hepatitis B vaccine: estimating cost-effectiveness using decision analysis. *Archives of pediatrics & adolescent medicine* 1999; **153**(2): 126-36.
6. Wong WW, Woo G, Heathcote EJ, Krahn M. Disease burden of chronic hepatitis B among immigrants in Canada. *Canadian journal of gastroenterology = Journal canadien de gastroenterologie* 2013; **27**(3): 137-47.
7. Fattovich G, Bortolotti F, Donato F. Natural history of chronic hepatitis B: special emphasis on disease progression and prognostic factors. *Journal of hepatology* 2008; **48**(2): 335-52.
8. Wen WH, Chang MH, Hsu HY, Ni YH, Chen HL. The development of hepatocellular carcinoma among prospectively followed children with chronic hepatitis B virus infection. *The Journal of pediatrics* 2004; **144**(3): 397-9.
9. Zhenhao J. [Cost-effectiveness analysis of neonatal hepatitis B immunization strategies in China]. *Xi'an： Xi'an Jiaotong University* 2012.
10. Hutton DW, Tan D, So SK, Brandeau ML. Cost-effectiveness of screening and vaccinating Asian and Pacific Islander adults for hepatitis B. *Annals of internal medicine* 2007; **147**(7): 460-9.
11. Siddiqui MR, Gay N, Edmunds WJ, Ramsay M. Economic evaluation of infant and adolescent hepatitis B vaccination in the UK. *Vaccine* 2011; **29**(3): 466-75.
12. Hutton DW, So SK, Brandeau ML. Cost-effectiveness of nationwide hepatitis B catch-up vaccination among children and adolescents in China. *Hepatology* 2010; **51**(2): 405-14.
13. Kanwal F, Gralnek IM, Martin P, Dulai GS, Farid M, Spiegel BM. Treatment alternatives for chronic hepatitis B virus infection: a cost-effectiveness analysis. *Annals of internal medicine* 2005; **142**(10): 821-31.
14. Wu B, Li T, Chen H, Shen J. Cost-effectiveness of nucleoside analog therapy for hepatitis B in China: a Markov analysis. *Value in health : the journal of the International Society for Pharmacoeconomics and Outcomes Research* 2010; **13**(5): 592-600.
15. Thiele M, Gluud LL, Fialla AD, Dahl EK, Krag A. Large variations in risk of hepatocellular carcinoma and mortality in treatment naive hepatitis B patients: systematic review with meta-analyses. *PloS one* 2014; **9**(9): e107177.
16. Liaw YF, Chu CM. Hepatitis B virus infection. *Lancet (London, England)* 2009; **373**(9663): 582-92.
17. Elgouhari HM, Abu-Rajab Tamimi TI, Carey WD. Hepatitis B virus infection: understanding its epidemiology, course, and diagnosis. *Cleveland Clinic journal of medicine* 2008; **75**(12): 881-9.

18. Liaw YF. Natural history of chronic hepatitis B virus infection and long-term outcome under treatment. *Liver international : official journal of the International Association for the Study of the Liver* 2009; **29 Suppl 1**: 100-7.
19. Fattovich G, Pantalena M, Zagni I, Realdi G, Schalm SW, Christensen E. Effect of hepatitis B and C virus infections on the natural history of compensated cirrhosis: a cohort study of 297 patients. *The American journal of gastroenterology* 2002; **97**(11): 2886-95.
20. Keeffe EB, Dieterich DT, Han SH, et al. A treatment algorithm for the management of chronic hepatitis B virus infection in the United States: 2008 update. *Clinical gastroenterology and hepatology : the official clinical practice journal of the American Gastroenterological Association* 2008; **6**(12): 1315-41; quiz 286.
21. McMahon BJ. The natural history of chronic hepatitis B virus infection. *Hepatology* 2009; **49**(5 Suppl): S45-55.
22. Nguyen VT, Law MG, Dore GJ. Hepatitis B-related hepatocellular carcinoma: epidemiological characteristics and disease burden. *Journal of viral hepatitis* 2009; **16**(7): 453-63.
23. Shepherd J, Jones J, Takeda A, Davidson P, Price A. Adefovir dipivoxil and pegylated interferon alfa-2a for the treatment of chronic hepatitis B: a systematic review and economic evaluation. *Health technology assessment (Winchester, England)* 2006; **10**(28): iii-iv, xi-xiv, 1-183.
24. Veldhuijzen IK, Toy M, Hahne SJ, et al. Screening and early treatment of migrants for chronic hepatitis B virus infection is cost-effective. *Gastroenterology* 2010; **138**(2): 522-30.
25. Ting-Tsung C, G GR, Robert dM, et al. A comparison of entecavir and lamivudine for HBeAg-positive chronic hepatitis B. *The New England Journal of Medicine* 2006; **354**(10).
26. Ching-Lung L, Daniel S, S LA, et al. Entecavir versus lamivudine for patients with HBeAg-negative chronic hepatitis B. *The New England Journal of Medicine* 2006; **354**(10).
27. Colonno RJ RR, Pokornowski K, et al. . Four-year assessment of entecavir resistance in nucleoside naïve and lamivudine refractory patients. . *J Hepatol* 2007; **46**(S294).
28. Wu CY, Lin JT, Ho HJ, et al. Association of nucleos(t)ide analogue therapy with reduced risk of hepatocellular carcinoma in patients with chronic hepatitis B: a nationwide cohort study. *Gastroenterology* 2014; **147**(1): 143-51.e5.
29. Xu Y, Zhang Y-G, Wang X, et al. Long-term antiviral efficacy of entecavir and liver histology improvement in Chinese patients with hepatitis B virus-related cirrhosis. *World J Gastroenterol* 2015; **21**(25): 7869-76.
30. Yan Wang TL, Zenglu Han, Yundong Qu, Chunlei Lin, Lei Wang, Baohua Yang. Incidence and predictors of hepatocellular carcinoma in Chinese hepatitis B virus-related cirrhotic patients receiving antiviral therapy: a retrospective cohort study. *International Journal of Clinical and Experimental Medicine* 2018; **11**(9).
31. Yip TC, Wong VW, Chan HL, Tse YK, Lui GC, Wong GL. Tenofovir Is Associated With Lower Risk of Hepatocellular Carcinoma Than Entecavir in Patients With Chronic HBV Infection in China. *Gastroenterology* 2020; **158**(1): 215-25.e6.
32. Tsai TY, Hung TH, Livneh H, Lin IH, Lu MC, Yeh CC. Chinese herbal medicine therapy and the risk of mortality for chronic hepatitis B patients with concurrent liver cirrhosis: a nationwide population-based cohort study. *Oncotarget* 2018; **9**(26): 18214-23.
33. Chang TT, Gish RG, de Man R, et al. A comparison of entecavir and lamivudine for HBeAg-positive chronic hepatitis B. *The New England journal of medicine* 2006; **354**(10): 1001-10.
34. Lai CL, Shouval D, Lok AS, et al. Entecavir versus lamivudine for patients with HBeAg-negative

chronic hepatitis B. *The New England journal of medicine* 2006; **354**(10): 1011-20.
35. Colonno RJ RR, Pokornowski K, et al. . Four-year assessment of entecavir resistance in nucleoside naïve and lamivudine refractory patients. . *Journal of hepatology* 2007; **46**(294).
36. Liaw YF, Sung JJ, Chow WC, et al. Lamivudine for patients with chronic hepatitis B and advanced liver disease. *The New England journal of medicine* 2004; **351**(15): 1521-31.
37. Organ Transplantation Branch，Chinese Medical Doctor Association; Chinese Society of Organ Transplantation，Chinese Medical Association. The Chinese clinical practice guideline on liver transplantation for hepatocellular carcinoma (2018). 2018.
38. Sun H-C, Xie L, Yang X-R, et al. Shanghai Score: A Prognostic and Adjuvant Treatment-evaluating System Constructed for Chinese Patients with Hepatocellular Carcinoma after Curative Resection. *Chin Med J (Engl)* 2017; **130**(22): 2650-60.
39. Toy M, Salomon JA, Jiang H, et al. Population health impact and cost-effectiveness of monitoring inactive chronic hepatitis B and treating eligible patients in Shanghai, China. *Hepatology* 2014; **60**(1): 46-55.
40. Levy AR, Kowdley KV, Iloeje U, et al. The impact of chronic hepatitis B on quality of life: a multinational study of utilities from infected and uninfected persons. *Value in health : the journal of the International Society for Pharmacoeconomics and Outcomes Research* 2008; **11**(3): 527-38.
41. Pwu RF, Chan KA. Cost-effectiveness analysis of interferon-alpha therapy in the treatment of chronic hepatitis B in Taiwan. *Journal of the Formosan Medical Association = Taiwan yi zhi* 2002; **101**(9): 632-41.
42. Lam ET, Lam CL, Lai CL, Yuen MF, Fong DY, So TM. Health-related quality of life of Southern Chinese with chronic hepatitis B infection. *Health and quality of life outcomes* 2009; **7**: 52.
43. Che YH, You J, Chongsuvivatwong V, et al. Dynamics and liver disease specific aspects of quality of life among patients with chronic liver disease in Yunnan, China. *Asian Pacific journal of cancer prevention : APJCP* 2014; **15**(12): 4765-71.
44. Jia YX, Cui FQ, Li L, et al. Comparison between the EQ-5D-5L and the EQ-5D-3L in patients with hepatitis B. *Quality of life research : an international journal of quality of life aspects of treatment, care and rehabilitation* 2014; **23**(8): 2355-63.
45. Zhuang G, Zhang M, Liu Y, et al. Significant impairment of health-related quality of life in mainland Chinese patients with chronic hepatitis B: a cross-sectional survey with pair-matched healthy controls. *Health and quality of life outcomes* 2014; **12**: 101.
46. Xiao Min WH, Zhang Wei, etc. Cost-effectiveness analysis of hepatitis B vaccination strategy for high risk adults in Beijing. *Chinese Journal of Disease Control & Prevention* 2015; **7**: 730-4.
47. Qian Y, Xiaoling F, Xi Z, Chenggang J. Evaluation on Life Quality Utility Weight of Hepatitis B Patients with Different Disease Status. *Chinese Health Economics* 2016; (6): 70.
48. Zhu D, Guo N, Wang J, et al. Socioeconomic inequality in Hepatitis B vaccination of rural adults in China. *Hum Vaccin Immunother* 2018; **14**(2): 464-70.
49. University of Hong Kong-Shenzhen Hospital [National lists of essential medicines]. Shenzhen, 2020.
50. Zhang S, Ma Q, Liang S, et al. Annual economic burden of hepatitis B virus-related diseases among hospitalized patients in twelve cities in China. *Journal of viral hepatitis* 2016; **23**(3): 202-10.
51. Yingzi Ming QZ, Baoren Tu, Gangcheng Kong, Hao Li, Ying Niu, Bo Peng, Junhui Li, Meng Yu and Min Yang. Liver Transplantation in China. 2018.
52. Wang Xin TH. Cost-effectiveness analysis of three immunosuppressive drugs in liver transplant

recipients. *China Pharmacy* 2005; **16**(15).
53. Amini A, Varsaneux O, Kelly H, et al. Diagnostic accuracy of tests to detect hepatitis B surface antigen: a systematic review of the literature and meta-analysis. *BMC infectious diseases* 2017; **17**(Suppl 1): 698.
54. Shivkumar S, Peeling R, Jafari Y, Joseph L, Pai NP. Rapid point-of-care first-line screening tests for hepatitis B infection: a meta-analysis of diagnostic accuracy (1980-2010). *The American journal of gastroenterology* 2012; **107**(9): 1306-13.
55. WHO. WHO guide to cost-eff ectiveness analysis. Geneva:World Health Organization, 2003.
56. Bill & Melinda Gates Foundation. Methods for economic evaluation project (MEEP): the Gates reference case. Seattle, WA: Bill & Melinda Gates Foundation, 2014.
57. Zhu D, Wang J, Wangen KR. Hepatitis B vaccination coverage rates among adults in rural China: are economic barriers relevant? *Vaccine* 2014; **32**(49): 6705-10.
58. Razavi-Shearer D, Gamkrelidze I, Nguyen MH, et al. Global prevalence, treatment, and prevention of hepatitis B virus infection in 2016: a modelling study. *The Lancet Gastroenterology & Hepatology* 2018; **3**(6): 383-403.
59. Fanqiang W, Quancheng Z, Fang W, Hongli W. Analysis of five quantitative test results of hepatitis B in 5739 pregnant women in Qinhuangdao. *Labeled Immunoassays and Clinical Medicine* 2014; **21**(06): 696-7+700.
60. Fengrui C, Simei F, Xin T, et al. Investigation and analysis on serum epidemiology of hepatitis B in different years in Jilin Province,China. *Chinese Journal of Biologicals* 2016; **29**(07): 725-8+32.
61. Shishen W, Yuhui T, Yuchun T, et al. Epidemiological study of hepatitis B and hepatitis C infections in Northeastern China and the beneficial effect of the vaccination strategy for hepatitis B: a cross-sectional study. *BMC Public Health* 2018; **18**(1).
62. Hao GY, Xing FD, Jin X, et al. The prevalence of hepatitis B infection in central China: An adult population-based serological survey of a large sample size. *Journal of Medical Virology* 2017; **89**(3).
63. Yonghao G, Jun L, Pumei D, et al. Serological survey of hepatitis B at national disease surveillance sites in Henan Province in 2014. *Chinese Journal of Preventive Medicine* 2016; **50**(03): 279-81.
64. Lin L, Zhengrong D, Yi K, et al. Serological Epidemiology Analysis of the Hepatitis B Virus Infection in Yunnan Province. *Chinese Journal of Vaccines and Immunization* 2011; **17**(01): 33-7.
65. Zhixin X, Shubo L, Kun G, et al. Investigation of Related Knowledge and Behavior of Viral Hepatitis among Food and Public Places Personnel in Changping District of Beijing City. *Occupation and Health* 2008; (21): 2298-9.
66. Zhang Y-q, Bian S-n, Liu X-q, et al. Positive Rate of Different Hepatitis B Virus Serological Markers in Peking Union Medical College Hospital,a General Tertiary Hospital in Beijing. *Chinese Medical Sciences Journal* 2016; **31**(01): 17-22.
67. Hongrui R. Detection results of hepatitis B two half-and-half among 4 039 cases of pre-pregnancy eugenics examination couples. *Occupation and Health* 2019; **35**(10): 1419-21.
68. Li Z, Aiqiang X, Bingyu Y, et al. Analysis on Hepatitis B Vaccine Coverage among the Population of 1 ~ 59 Years Old in Shandong Province. *Chinese Journal of Vaccines and Immunization* 2009; **15**(02): 159-62.
69. Yanyan W, Wei Z, Zheng Z, et al. Community-based epidemiological survey of adult hepatitis B in Chaoyang District, Beijing. *Chinese Journal of Epidemiology* 2015; **36**(10): 1104-8.
70. Fuqiang C, Xiaohong G, Yuansheng C, et al. Vaccination Progress of Hepatitis B Vaccine and

Epidemiology Changes of Carrying Rate of Hepatitis B Surface Antigen by Province in China, 1992-2006. *Chinese Journal of Vaccines and Immunization* 2012; **18**(01): 6-13.
71. Haiyan H, Ying Z, Weishen W, Chao L, Ailan S. Seroepidemiology of hepatitis B in healthy population in Tianjin, 2010. *Disease Surveillance* 2011; **26**(12): 939-42.
72. Fanqiang W, Quancheng Z, Yaqi L. Analysis of Quantitative Test Results of Five Indexes of Hepatitis B for 10499 Different Cases in Qinhuangdao. *Labeled Immunoassays and Clinical Medicine* 2015; **22**(04): 310-2+6.
73. Dingyu M, Wenyong H, Jingyuan Y, et al. Prevalence pattern of hepatitis B among minority population in Guizhou Province. *Modern Preventive Medicine* 2016; **043**(001): 11-4,48.
74. Yuling L, Jichun W. Infection Status Survey of Hepatitis B Virus in Shenyang Citizens. *Journal of Microbiology* 2012; **32**(04): 103-5.
75. Yujie M, Fang B, Jianing S, Zhaodan S, Crane Y, Xue Z. Seroepidemiological analysis of viral hepatitis B among healthy people in Heilongjiang Province. *Chinese Journal of Public Health* 2017; **33**(10): 1485-8.
76. Shihong Y, Dan X, Yinan H, Yue Y. Analysis on the distribution characteristics of hepatitis B surface antigen antibodies in the population of 0-60 years old in Dalian. *Journal of Medical Pest Control* 2015; **31**(09): 1045-6+9.
77. Hong R, Xin Z, Jian L, Yanting L, Yang S, Jiayu H. Seroepidemiological analysis on hepatitis B virus infection among community residents in Shanghai. *Chinese Journal of Public Health* 2013; **29**(11): 1568-71.
78. Fangyan S, Ziyi J, Feng L. Sampling survey of hepatitis B seroepidemiology among people over 1 year old in Jiangyin City. *Practical Preventive Medicine* 2015; **22**(03): 328-30.
79. Juan W, Yonghai D, Fuyang G, et al. A prevalence and knowledge survey on hepatitis B among population in rural areas in a city of Anhui Province. *Chinese Journal of Disease Control & Prevention* 2010; **14**(08): 697-700.
80. Lifang H, Yong Z, Xiuhui Y, Dongjuan Z. Epidemiological characteristics of hepatitis B infection among people aged 1-29 years in Fujian Province. *Strait Journal of Preventive Medicine* 2018; **24**(03): 32-3.
81. Wanshen G, Qian L, Yonghao G, et al. Seroepidemiological survey of adult viral hepatitis B in Henan Province in 2012. *Journal of Zhengzhou University(Medical Sciences)* 2014; **49**(01): 53-9.
82. Jun L, Qing C, Jie L, willow, Yang H. Seroepidemiological survey of hepatitis B in couples planning pregnancy in Chongqing in 2013. *Chinese Journal of Clinical Infectious Diseases* 2014; **7**(06): 506-10.
83. Haijun L, essential T, Yuanyuan Y, et al. Analysis of the current status of carrying hepatitis B surface antigen in the population of Ningxia in 2010. *Ningxia Medical Journal* 2014; **36**(01): 78-80.
84. Cui F, Shen L, Li L, et al. Prevention of Chronic Hepatitis B after 3 Decades of Escalating Vaccination Policy, China. *Emerging Infectious Disease journal* 2017; **23**(5): 765.

<u>**Consolidated Health Economic Evaluation Reporting Standards (CHEERS) Checklist**</u>

Items to include when reporting economic evaluations of health interventions

The **ISPOR CHEERS Task Force Report**, *Consolidated Health Economic Evaluation Reporting Standards (CHEERS)—Explanation and Elaboration: A Report of the ISPOR Health Economic Evaluations Publication Guidelines Good Reporting Practices Task Force*, provides examples and further discussion of the 24-item CHEERS Checklist and the CHEERS Statement. It may be accessed via the *Value in Health* or via the ISPOR Health Economic Evaluation Publication Guidelines – CHEERS: Good Reporting Practices webpage: http://www.ispor.org/TaskForces/EconomicPubGuidelines.asp

**Section**	**Item No**	**Recommendation**	**Reported on page No/line No**
**Title and Abstract**			
Title	1	Identify the study as an economic evaluation or use more specific terms such as "cost-effectiveness analysis", and describe the interventions compared.	1
Abstract	2	Provide a structured summary of objectives, perspective, setting, methods (including study design and inputs), results (including base case and uncertainty analyses), and conclusions.	3
**Introduction**			
Background and objectives	3	Provide an explicit statement of the broader context for the study. Present the study question and its relevance for health policy or practice decisions.	5
**Methods**			
Target population and subgroups	4	Describe characteristics of the base case population and subgroups analysed, including why they were chosen.	6
Setting and location	5	State relevant aspects of the system(s) in which the decision(s) need(s) to be made.	6
Study perspective	6	Describe the perspective of the study and relate this to the costs being evaluated.	6
Comparators	7	Describe the interventions or strategies being compared and state why they were chosen.	7
Time horizon	8	State the time horizon(s) over which costs and consequences are being evaluated and say why appropriate.	6-7

Discount rate	9	Report the choice of discount rate(s) used for costs and outcomes and say why appropriate.	8
Choice of health outcomes	10	Describe what outcomes were used as the measure(s) of benefit in the evaluation and their relevance for the type of analysis performed.	7-8
Measurement of effectiveness	11a	*Single study-based estimates*: Describe fully the design features of the single effectiveness study and why the single study was a sufficient source of clinical effectiveness data.	N/A
	11b	*Synthesis-based estimates*: Describe fully the methods used for identification of included studies and synthesis of clinical effectiveness data.	6-7<br>Figure S2
Measurement and valuation of preference based outcomes	12	If applicable, describe the population and methods used to elicit preferences for outcomes.	8
Estimating resources and costs	13a	*Single study-based economic evaluation*: Describe approaches used to estimate resource use associated with the alternative interventions. Describe primary or secondary research methods for valuing each resource item in terms of its unit cost. Describe any adjustments made to approximate to opportunity costs.	N/A
	13b	*Model-based economic evaluation*: Describe approaches and data sources used to estimate resource use associated with model health states. Describe primary or secondary research methods for valuing each resource item in terms of its unit cost. Describe any adjustments made to approximate to opportunity costs.	7
Currency, price date, and conversion	14	Report the dates of the estimated resource quantities and unit costs. Describe methods for adjusting estimated unit costs to the year of reported costs if necessary. Describe methods for converting costs into a common currency base and the exchange rate.	6<br>Table 1
Choice of model	15	Describe and give reasons for the specific type of decision-analytical model used. Providing a figure to show model structure is strongly recommended.	6<br>Figure S1
Assumptions	16	Describe all structural or other assumptions underpinning the decision-analytical model.	6−8
Analytical methods	17	Describe all analytical methods supporting the	6−8

		evaluation. This could include methods for dealing with skewed, missing, or censored data; extrapolation methods; methods for pooling data; approaches to validate or make adjustments (such as half cycle corrections) to a model; and methods for handling population heterogeneity and uncertainty.	
**Results**			
Study parameters	18	Report the values, ranges, references, and, if used, probability distributions for all parameters. Report reasons or sources for distributions used to represent uncertainty where appropriate. Providing a table to show the input values is strongly recommended.	Table 1<br>Figure S2
Incremental costs and outcomes	19	For each intervention, report mean values for the main categories of estimated costs and outcomes of interest, as well as mean differences between the comparator groups. If applicable, report incremental cost-effectiveness ratios.	8-10<br>Figure 1<br>Table S1
Characterizing uncertainty	20a	*Single study-based economic evaluation*: Describe the effects of sampling uncertainty for the estimated incremental cost and incremental effectiveness parameters, together with the impact of methodological assumptions (such as discount rate, study perspective).	N/A
	20b	*Model-based economic evaluation*: Describe the effects on the results of uncertainty for all input parameters, and uncertainty related to the structure of the model and assumptions.	10<br>Figure 2<br>Figure S3
Characterizing heterogeneity	21	If applicable, report differences in costs, outcomes, or cost-effectiveness that can be explained by variations between subgroups of patients with different baseline characteristics or other observed variability in effects that are not reducible by more information.	9-10<br>Table 2
**Discussion**			
Study findings, limitations, generalizability, and current knowledge	22	Summarise key study findings and describe how they support the conclusions reached. Discuss limitations and the generalisability of the findings and how the findings fit with current knowledge.	10−12
**Other**			
Source of funding	23	Describe how the study was funded and the role of the funder in the identification, design, conduct, and reporting of the analysis. Describe other non-monetary	2

		sources of support.	
Conflicts of interest	24	Describe any potential for conflict of interest of study contributors in accordance with journal policy. In the absence of a journal policy, we recommend authors comply with International Committee of Medical Journal Editors recommendations.	2

For consistency, the CHEERS Statement checklist format is based on the format of the CONSORT statement checklist

The **ISPOR CHEERS Task Force Report** provides examples and further discussion of the 24-item CHEERS Checklist and the CHEERS Statement. It may be accessed via the *Value in Health* link or via the ISPOR Health Economic Evaluation Publication Guidelines – CHEERS: Good Reporting Practices webpage: http://www.ispor.org/TaskForces/EconomicPubGuidelines.asp

The citation for the CHEERS Task Force Report is: Husereau D, Drummond M, Petrou S, et al. Consolidated health economic evaluation reporting standards (CHEERS)—Explanation and elaboration: A report of the ISPOR health economic evaluations publication guidelines good reporting practices task force. *Value Health* 2013; 16:231–50.
